# Focused navigation for respiratory–motion-corrected free-running radial 4D flow MRI

**DOI:** 10.1002/mrm.29634

**Published:** 2023-03-06

**Authors:** Mariana B. L. Falcão, Giulia M. C. Rossi, Tobias Rutz, Milan Prša, Estelle Tenisch, Liliana Ma, Elizabeth K. Weiss, Justin J. Baraboo, Jérôme Yerly, Michael Markl, Matthias Stuber, Christopher W. Roy

**Affiliations:** 1 Department of Diagnostic and Interventional Radiology, Lausanne University Hospital (CHUV) and University of Lausanne (UNIL), Lausanne, Switzerland; 2 Service of Cardiology, Centre de Resonance Magnétique Cardiaque (CRMC), Lausanne University Hospital and University of Lausanne, Lausanne, Switzerland; 3 Woman-Mother-Child Department, Lausanne University Hospital and University of Lausanne, Lausanne, Switzerland; 4 Department of Radiology, Feinberg School of Medicine, Northwestern University, Chicago, Illinois USA; 5 Department of Biomedical Engineering, Northwestern University, Chicago, Illinois USA; 6 Center for Biomedical Imaging (CIBM), Lausanne, Switzerland

**Keywords:** 4D flow MRI, fNAV, focused navigation, free-running 3D radial PC-MRI, motion correction

## Abstract

**Purpose:**

To validate a respiratory motion correction method called focused navigation (fNAV) for free-running radial whole-heart 4D flow MRI.

**Methods:**

Using fNAV, respiratory signals derived from radial readouts are converted into three orthogonal displacements, which are then used to correct respiratory motion in 4D flow datasets. Hundred 4D flow acquisitions were simulated with non-rigid respiratory motion and used for validation. The difference between generated and fNAV displacement coefficients was calculated. Vessel area and flow measurements from 4D flow reconstructions with (fNAV) and without (uncorrected) motion correction were compared to the motion-free ground-truth. In 25 patients, the same measurements were compared between fNAV 4D flow, 2D flow, navigator-gated Cartesian 4D flow, and uncorrected 4D flow datasets.

**Results:**

For simulated data, the average difference between generated and fNAV displacement coefficients was 0.04 ± 0.32 mm and 0.31 ± 0.35 mm in the x and y directions, respectively. In the z direction, this difference was region-dependent (0.02 ± 0.51 mm up to 5.85 ± 3.41 mm). For all measurements (vessel area, net volume, and peak flow), the average difference from ground truth was higher for uncorrected 4D flow datasets (0.32 ± 0.11 cm^2^, 11.1 ± 3.5 mL, and 22.3 ± 6.0 mL/s) than for fNAV 4D flow datasets (0.10 ± 0.03 cm^2^, 2.6 ± 0.7 mL, and 5.1 ±0.9 mL/s, *p* < 0.05). In vivo, average vessel area measurements were 4.92 ± 2.95 cm^2^, 5.06 ± 2.64 cm^2^, 4.87 ± 2.57 cm^2^, 4.87 ± 2.69 cm^2^, for 2D flow and fNAV, navigator-gated and uncorrected 4D flow datasets, respectively. In the ascending aorta, all 4D flow datasets except for the fNAV reconstruction had significantly different vessel area measurements from 2D flow. Overall, 2D flow datasets demonstrated the strongest correlation to fNAV 4D flow for both net volume (*r*^2^ = 0.92) and peak flow (*r*^2^ = 0.94), followed by navigator-gated 4D flow (*r*^2^ = 0.83 and *r*^2^ = 0.86, respectively), and uncorrected 4D flow (*r*^2^ = 0.69 and *r*^2^ = 0.86, respectively).

**Conclusion:**

FNAV corrected respiratory motion in vitro and in vivo, resulting in fNAV 4D flow measurements that are comparable to those derived from 2D flow and navigator-gated Cartesian 4D flow datasets, with improvements over those from uncorrected 4D flow.

## 1 ∣ INTRODUCTION

Three-dimensional phase-contrast (PC) MRI or “4D flow MRI” permits a quantitative evaluation of blood flowing throughout the heart and vessels.^1-5^ Increasingly, 4D flow MRI is used in the clinical assessment of heart disease,^6-8^ but despite advances in 4D flow imaging,^9-18^ the achievable volumetric coverage, ease-of-use, and acquisition time remain largely constrained by the need to compensate for respiratory motion. Conventional 4D flow acquisitions use a Cartesian sampling trajectory and a 1D navigator echo, prescribed along the lung liver interface, to limit data collection to a manually defined acceptance window during the end-expiratory respiratory phase.^19,20^ Therefore, acquisition efficiency is highly driven by the breathing pattern of each patient, leading to unpredictable scan times. In practice, 4D flow MRI can be acquired without navigators and throughout the respiratory cycle to improve efficiency or reduce scan times, but without additional respiratory compensation there will be a decrease in the accuracy of flow measurements and overall visualization of the vessels of interest.^21,22^

Recently, a respiratory-motion correction technique called focused navigation (fNAV), which estimates displacement from the data itself without the need for additional hardware, was developed for electrocardiogram (ECG)-triggered radial whole-heart cardiac magnetic resonance angiography (CMRA).^23^ Using fNAV, a 1D respiratory signal derived from a periodically sampled readout is converted into respiratory displacement measurements that span the entire acquisition along all three spatial dimensions using an auto-focusing algorithm.^24,25^ In this way, data can be used from the entire respiratory cycle while minimizing motion related artifacts, therefore, providing a more efficient acquisition and reconstruction of free-breathing data when compared to the conventional 1D navigator approach.

The goal of this work is to extend the fNAV methodology to a free-running 3D radial whole-heart PC-MRI acquisition.^10,26-28^ The use of fNAV for respiratory motion correction presents several potential advantages for free-running 3D radial PC-MRI. First, it does not require any additional scans to calibrate respiratory displacement.^29^ Second, it is capable of correcting motion for each individual readout, which generally cannot be done using motion correction methods that must first bin the data into respiratory phases.^30^ Finally, the 1D respiratory signal is readily derived from the radial imaging data itself and therefore, does not require significant modification^17^ or interruption^31^ of the imaging sequence to produce navigator data.

In this work, we adapted the fNAV methodology for use in the reconstruction of free-running 3D radial PC-MRI data, resulting in 4D flow images, hereafter, referred to as fNAV 4D flow datasets. We performed a comprehensive validation study in a numerical simulation based on data from a programmable pulsatile flow phantom, wherein displacement due to respiratory motion was simulated retrospectively. We, then, demonstrated the feasibility of this approach in a diverse cohort of patients with congenital heart disease (CHD). We tested the hypotheses that, first, fNAV can estimate and correct for the translational displacement of the heart because of respiration, and second, that fNAV 4D flow datasets produce comparable flow measurements to those from separately acquired reference standard 2D flow datasets and with a reduction in bias when compared to those derived from 4D flow datasets reconstructed without motion correction. We compared the vessel area, net volume, and peak flow measurements from fNAV 4D flow datasets to those from uncorrected 4D flow reconstructions of the same data, as well as to conventional navigator-gated Cartesian 4D flow MRI and to reference standard 2D flow MRI.

## 2 ∣ METHODS

### 2.1 ∣ fNAV 4D flow framework

**FIGURE 1:**
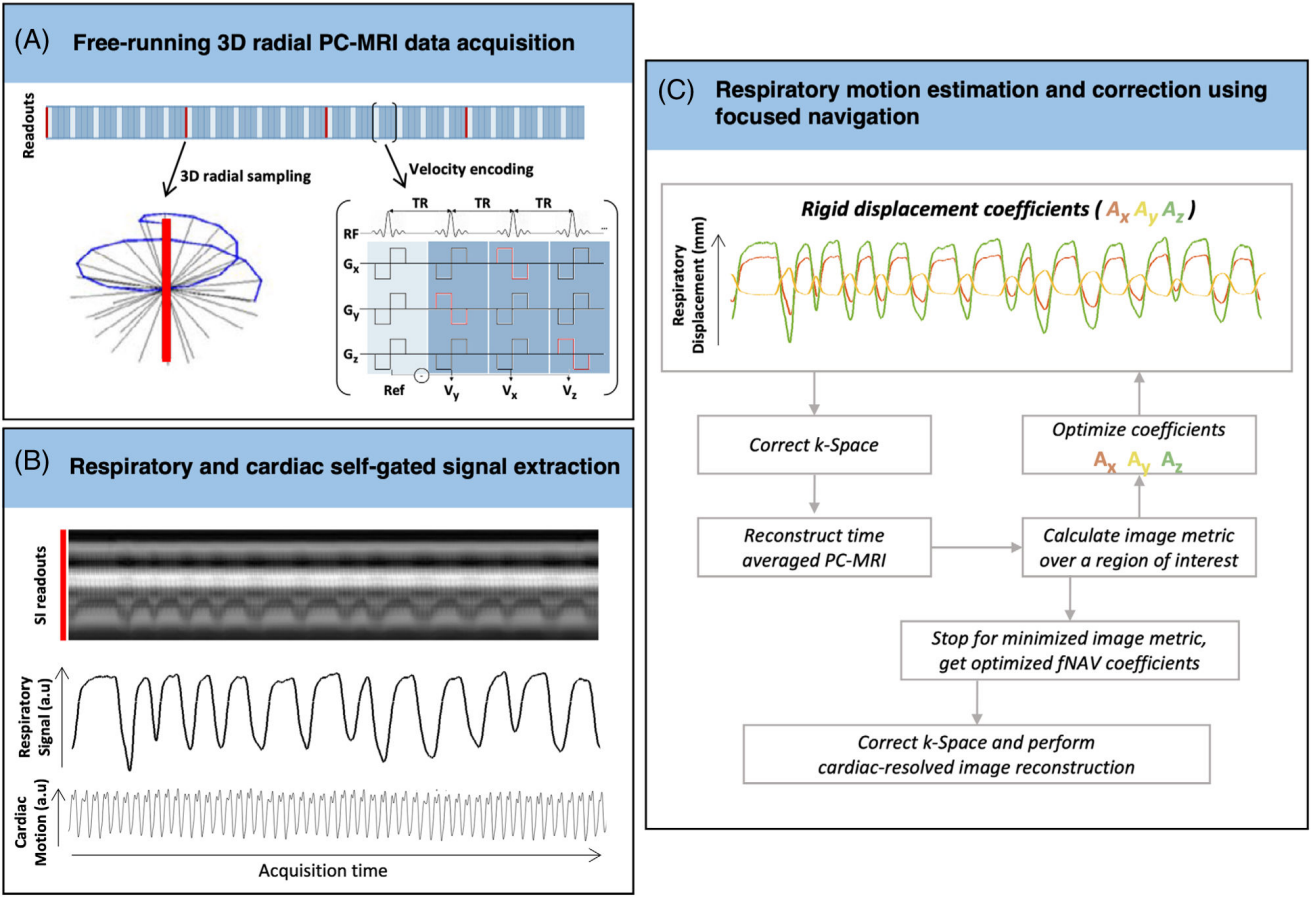
Summary of the focused navigation (fNAV) 4D flow pipeline. (A) From continuously acquired free-running 3D radial phase-contrast (PC)-MRI data, (B) respiratory and cardiac motion signals are extracted using self-gating. (C) Using the 1D respiratory self-gating curve, the displacement of the heart because of respiration along all three spatial dimensions was modeled as the product of the respiratory curve and three initially unknown fNAV coefficients (A_*x*_, A_*y*_, A_*z*_). The 3D translational motion determined by the product of the respiratory curve and fNAV coefficients was applied to the acquired k-space. The coefficients were then iteratively adjusted to minimize a metric based on the entropy of the image. The final optimized fNAV coefficients were used to correct the k-space data, which was reconstructed using a k-t sparse SENSE algorithm.^47^

A schematic overview of the proposed pipeline for the reconstruction of fNAV 4D flow datasets is shown in Figure 1. Free-running 3D radial PC-MRI data were acquired (Figure 1A) as previously described,^27^ wherein readouts followed a spiral phyllotaxis sampling pattern with each interleave rotated by the golden angle relative to the previous one.^32-34^ At the beginning of each interleave, one readout oriented along the superior–inferior (SI) direction was acquired for subsequent extraction of respiratory and cardiac self-gating signals (Figure 1B).^28,34^ The SI readout is only acquired for one velocity encode, whereas the imaging readouts were repeated four times for balanced four-point velocity encoding.^27,28^

To reconstruct the acquired data, the fNAV framework, previously developed for ECG-triggered 3D radial CMRA, was adapted for free-running 3D radial PC-MRI with the following four steps. First, a unitless respiratory curve was extracted from the concatenated SI readouts using principal component analysis.^28,34^ Second, the respiratory-dependent displacement of the heart in millimeters was modeled along all three spatial dimensions, by multiplying the extracted respiratory curve with three fNAV coefficients describing the maximum amplitude of respiratory motion in three orthogonal directions (A_*x*_, A_*y*_, A_*z*_).

The goal of fNAV is to find the optimal coefficients (A_*x*_, A_*y*_, A_*z*_) that best represent the displacement in each dataset. Therefore, in a third step, the fNAV coefficients were iteratively estimated: the product of the respiratory curve and fNAV coefficients was applied as a phase shift to the acquired k-space data (Figure 1C), an intermediate 4D flow dataset with the current translational motion correction was reconstructed using a non-uniform Fourier transform, and a time-averaged phase-contrast magnetic resonance angiogram (PC-MRA) was calculated by multiplying the sum of squares of the magnitude and phase images. Next, a metric for blur (entropy of the gradient image) was calculated over a region of interest (ROI) automatically placed at the approximate center of the heart, by calculating the center of mass of the PC-MRA^23,25^ and used to update the coefficients. The phase shifts corresponding to the optimized fNAV coefficients that minimize the image metric were applied to the acquired k-space.

In addition to correcting respiratory motion, cardiac self-gating was used as previously described.^27,28,34^ Notably, the uninterrupted gradient echo sequence that the 3D radial PC-MRI sequence is based on has been shown to interfere with the ECG signal and therefore, self-gating has been shown to be more reliable.^35^ Finally, a k-t sparse SENSE algorithm^27,28^ was used to reconstruct the final fNAV 4D flow images with corrected respiratory motion and resolved cardiac motion (x-y-z-cardiac-velocity dimensions). After first normalizing each acquisition by the maximum signal from a gridded image reconstruction, regularization parameters for reconstructing the fNAV 4D flow datasets were 0.0075 for total variation applied along the cardiac dimension and 0.015 for total variation applied along the spatial dimension.^28^

### 2.2 ∣ Optimization of fNAV coefficients

The PC-MRA, as described above, was chosen in place of the magnitude images used in the original fNAV study as it provided separation between the cardiac anatomy and background allowing for an automated ROI selection as described above, and improved detection of rigid translational movement of the heart without being impacted by non-cardiac anatomy. The size of the ROI was empirically chosen to be one third the acquisition field of view (FOV), which in general provided adequate coverage of the heart.

The entropy of the gradient image (H) was chosen as a blur metric based on previous work and is described by the following^23^:

$$\;H=-\sum_{x}\sum_{y}\sum_{z}p_{\text{xyz}}\;\log_{2}\;(p_{\text{xyz}})\;,\;\;p_{\text{xyz}}=\frac{g_{\text{xyz}}}{\sum_{\text{xyz}}g_{\text{xyz}}},\text{and}\;g_{\text{xyz}}=\sqrt{|\nabla_{x}I{|}^{2}+|\nabla_{y}I{|}^{2}+|\nabla_{z}I{|}^{2}}$$

where (*x*, *y*, *z*) define a 3D region, (*p*) is the normalized voxel intensity from the gradient (*g*) of the intermediate image (I), and ∇ is approximated by 1D finite differences.^17,25,36,37^ Optimized fNAV coefficients were found using a steepest descent algorithm where the gradient of H as a function of the fNAV coefficients was approximated numerically.^23^

### 2.3 ∣ Validation using a numerical simulation

#### 2.3.1 ∣ In vitro acquisitions

An MRI-compatible pulsatile flow pump (delivering flow rates of ~250 mL/s) connected to a U-shaped polyvinyl-chloride pipe, representing a simplified aorta model,^38^ was scanned on a 1.5 MAGNETOM Aera scanner (Siemens Healthcare, Erlangen, Germany). The U-pipe contained a section with variable diameter to mimic stenosis, and as a result to create non-uniform flow profiles. The cardiac frequency simulated was ~60 cycles per minute (~1 Hz). To simulate a contrast-enhanced flow scan, gadolinium enhanced water was used as fluid. A fully sampled conventional navigator-gated Cartesian 4D flow sequence was acquired with the following scan parameters: TE = 2.3 ms, TR = 5.1 ms, 15° RF excitation angle, FOV = 54 × 351.5 × 370 mm^3^, base resolution = 24 × 152 × 160, isotropic spatial resolution = (2.3 mm)^3^, maximum velocity encoded = 150 cm/s, total scan time of 8.1 min.

#### 2.3.2 ∣ Generating simulated data

**FIGURE 2:**
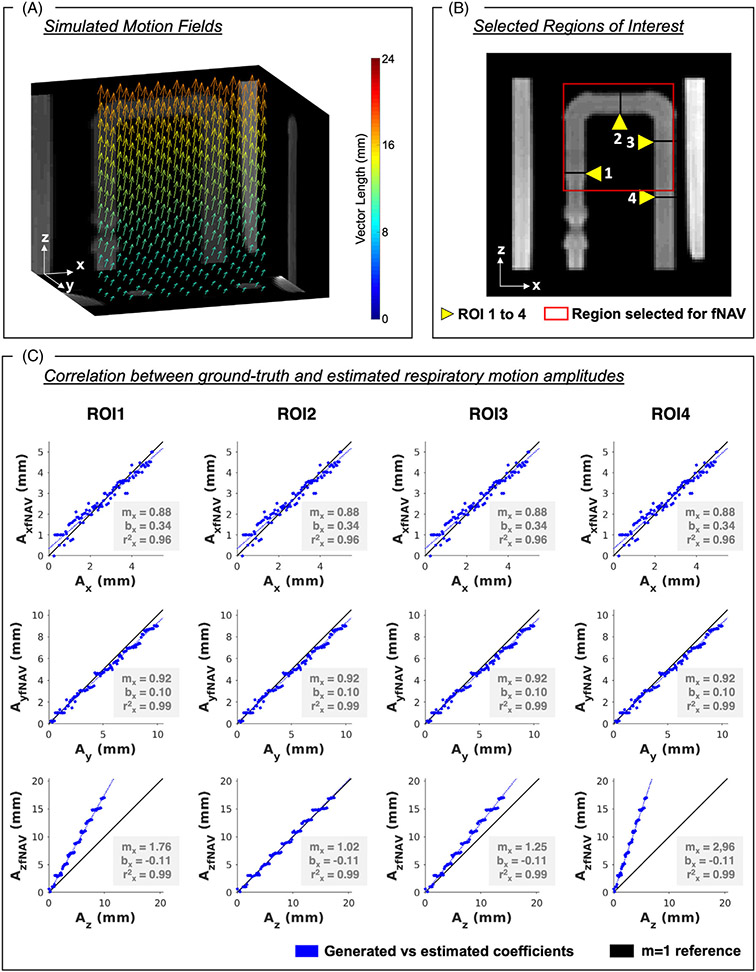
Phantom validation of focused navigation (fNAV) for rigid respiratory motion correction. Using the fNAV framework, 3D fNAV coefficients were estimated for each translational displacement of 100 simulated datasets. Vector length describes the 3D direction and amplitude of the voxel-wise motion field. Displacement along the z-direction was simulated to be larger at the top of the phantom and smaller at the bottom. (A) The 3D figure shows the motion fields oriented from top to bottom (B) For four regions of interest (ROI), (C) the correlation between the generated coefficients (A_*x*_, A_*y*_, A_*z*_) and the estimated coefficients (A_xfNAV_, A_yfNAV_, A_zfNAV_) was strong for the three directions of displacement (*r*^2^ > 0.9). ROI 3 was the region with the best 1–1 correlation between A_z_ and A_zfNAV_, with differences between A_z_ and A_zfNAV_ increasing with the distance from ROI 3. Yellow arrows point to the ROIs used for this analysis. The red square marks the region chosen for the fNAV correction. The blue lines describe regression lines, and black lines show the identity line. m, slope; b, intercept; ρ, Pearson correlation coefficient.

To mimic the effects of realistic respiratory motion on our phantom data, a non-rigid deformation field was modeled with uniform displacement along the x and y directions, but linearly increasing displacement along the z direction (Figure 2A). Each deformation field was multiplied by a time-varying respiratory curve modeled from a healthy volunteer acquisition with approximate respiratory peak frequency of ~0.15 Hz. We, then, generated simulated 3D radial k-space readouts after applying the deformation field to the in vitro images. The 3D radial k-space readouts were simulated to match the same sampling scheme and undersampling factor from the in vivo acquisitions (4820 spiral interleaves and 21 radial readouts per interleave, see in vivo section). A total of 100 unique variations of the maximum 3D displacement for each direction (A_*x*_: 0–5 mm, A_*y*_: 0–10 mm, A_*z*_: 0–20 mm) were generated using this pipeline, resulting in 100 corresponding simulated “free-breathing” radial 4D flow acquisitions.^23^

#### 2.3.3 ∣ Accuracy of respiratory motion correction

Using the fNAV 4D flow framework described above, a 3D region of interest was manually selected (fNAV region, Figure 2B), and estimated 3D displacements (fNAV coefficients) were obtained for all 100 simulated 4D flow datasets. Four 2D ROIs were manually selected. The error between ground-truth simulated displacement and fNAV coefficients for each ROI was quantified in terms of mean and SD, as well as using linear regression and Pearson correlation measures.

#### 2.3.4 ∣ Influence of respiratory motion

To study the influence of respiratory motion on both image quality and flow quantification, a subset (*n* = 20) of the simulated acquisitions were chosen such that they spanned the range of the previously generated maximum translational displacements and used for 4D flow image reconstruction, both with correction from the estimated fNAV coefficients (fNAV 4D flow) and without motion correction (uncorrected 4D flow). Additionally, the simulated 4D flow data that did not include any displacement because of respiratory motion, was reconstructed and used as the ground-truth reference.^21^

For each of the four ROIs described above, the vessel area was used as a surrogate measure of blur because of respiratory motion and was calculated as the sum of all voxels included in the ROI, multiplied by the area of each voxel. The vessel area was calculated for the ground-truth dataset, as well as for all uncorrected and fNAV 4D flow datasets. The mean and SD of the vessel area for each ROI was calculated across the entire range of displacements reconstructed (*n* = 20), for uncorrected and fNAV 4D flow datasets separately. The difference of vessel area from the ground-truth was also compared between the two groups.

For each reconstructed dataset and for each ROI, net volume and peak flow measurements were compared with the ground-truth reference. Mean and SD measures were calculated for each flow measurement and for each ROI. The differences from the ground-truth were calculated for uncorrected and fNAV 4D flow datasets.

### 2.4 ∣ Feasibility in a cohort of CHD patients

#### 2.4.1 ∣ In vivo acquisitions

A cohort of 25 CHD patients (age, 7–60 years, 12 female) was included in this study (see Table S1 for demographics). Each study participant or their legal guardian provided written informed consent compliant with institutional guidelines. All images were acquired on a 1.5 T Magnetom Sola (Siemens Healthcare, Erlangen, Germany).

Patients underwent a gadolinium-enhanced (Gadobutrol, Bayer, Switzerland, 0.4 mmol/kg) MRI protocol for CHD, that included three standard 2D flow acquisitions placed on the ascending aorta (AAo), descending aorta (DAo), and main pulmonary artery (MPA). Sequence parameters for the 2D flow acquisitions were as follows: TE = 3.0–3.6 ms, TR = 5.25–6.14 ms, 20° RF excitation angle, FOV = (136–234) × (288–340) mm^2^, base resolution = (132–136) × (192–288), spatial resolution = (1.0–1.8) × (1.0–1.8) × (4.0–6.0) mm^3^, velocity encoding = 150–200 cm/s. Additionally, a standard Cartesian 4D flow acquisition with respiratory navigation (nav-gated 4D flow) was included to obtain a reference 4D flow dataset. Sequence parameters were the following: TE = 2.2–2.3 ms, TR = 4.6–5.1 ms, 7–12° RF excitation angle, FOV = (125–258) × (188–308) × (200–400) mm^3^, base resolution = (50–103) × (75–123) × (96–160) isotropic spatial resolution = (2.1 mm)^3^–(2.5 mm)^3^, maximum velocity encoding = 150–200 cm/s, total scan time of 1.8–11.5 min.

At the end of each clinical exam, a free-running 3D radial PC-MRI sequence (Figure 1A) was acquired. Sequence parameters were as follows: TE = 2.9–3.1 ms, TR = 4.7–4.9 ms, 7° RF excitation angle, FOV = (220 mm)^3^–(240 mm)^3^, base resolution = (96)^3^ isotropic spatial resolution = (2.3 mm)^3^ – (2.5 mm)^3^, maximum velocity encoding = 150–200 cm/s, total scan time of 7.93–8.23 min, 4820 spiral interleaves and 21 radial readouts per interleave (1 SI + [5 readouts × 4-point velocity encoding]). Differences in scan time were because of changes in velocity encoding and FOV.

#### 2.4.2 ∣ Four dimensional flow image reconstruction

Free-running 3D radial PC-MRI acquisitions from all patients were reconstructed into fNAV 4D flow datasets, as described in Figure 1, and into 4D flow datasets without any type of respiratory compensation (uncorrected 4D flow). For fNAV 4D flow, the respiratory motion amplitudes estimated from fNAV were recorded and used for motion correction. Next, to reconstruct fNAV and uncorrected 4D flow datasets, data were binned into 17 to 30 cardiac phases (temporal resolution = 23.8–42.2 ms) and reconstructed using the k-t sparse SENSE algorithm described previously.^27,28^ Spatial resolution of the reconstructed datasets matched the acquired spatial resolution, (2.3 mm)^3^–(2.5 mm)^3^. All reconstructions of fNAV and uncorrected 4D flow datasets were performed in MATLAB (The MathWorks, Natick, MA) and took on average 2.8 ± 0.3 h to reconstruct including 2.5 min for fNAV. The nav-gated 4D flow datasets were reconstructed at the scanner during the examination using the vendor-provided reconstruction pipeline for these datasets. Streamlines derived from fNAV, nav-gated and uncorrected 4D flow datasets were visually compared.

#### 2.4.3 ∣ Comparison of vessel area and flow measurements

The 2D flow and three 4D flow datasets (i.e., fNAV 4D flow, nav-gated 4D flow, and uncorrected 4D flow) were segmented and flow measurements were analyzed using cvi42, Circle (Calgary, AB, Canada). The flow analysis was performed by one observer that was not blinded to the type of data being analyzed. To assess and compare the accuracy of flow measurements from fNAV 4D flow relative to 2D flow and to the remaining 4D flow datasets, vessel planes were manually placed to match the same location of the 2D flow acquisitions covering the AAo, DAo, and MPA. Similarly to in vitro, the vessel area was used as a surrogate metric for blur because of respiratory motion and was compared between the 2D flow datasets and fNAV, nav-gated, and uncorrected 4D flow datasets. Net volume and peak flow measurements were calculated for every 4D/2D flow dataset. Four dimensional flow measurements (fNAV, nav-gated, and uncorrected) were compared to their analogous 2D flow measurement.

### 2.5 ∣ Statistical analysis

Each group of measurements was tested for normality using a Lilliefors test. In case of rejection of the null hypothesis at the 5% significance level, measures were compared using a nonparametric Wilcoxon signed rank test. Otherwise, a paired *t* test was used to compare the similarity between datasets. For the patient cohort, differences in net volume and peak flow measurements between 2D flow and 4D flow datasets (fNAV, nav-gated, and uncorrected) were estimated using Bland-Altman analysis. Additionally, the correlation between 2D flow measurements and measurements from all 4D flow datasets was calculated using linear regression and Pearson correlation measures, and the significance level of the correlation was given as a *p*-value, from testing the hypothesis of no correlation.

## 3 ∣ RESULTS

### 3.1 ∣ Validation in a pulsatile flow phantom

#### 3.1.1 ∣ Accuracy of respiratory motion correction

The fNAV 4D flow framework was able to estimate displacement because of respiratory motion in all 100 simulated 4D flow acquisitions. The error (mean and SD) in x and y directions was consistent across all four ROIs (A_*x*_–A_xfNAV_ = 0.04 ± 0.32 mm, A_y_–A_yfNAV_ = −0.31 ± 0.35 mm) because of the uniform displacement generated in those directions. However, the error in the z direction was greater in ROIs where the true non-rigid displacement diverges from the rigid translational correction provided by fNAV, yielding excellent agreement for ROI 2 (A_z_-A_zfNAV_ = 0.02 ± 0.51 mm), followed by increasing average error for ROI 3 (A_z_–A_zfNAV_ = 1.71 ± 1.16 mm), ROI 1 (A_z_–A_zfNAV_ = 3.78 ± 2.26 mm) and finally ROI 4 (A_z_–A_zfNAv_ = 5.85 ± 3.41 mm).

The linear regression measurements calculated between the generated displacements and those estimated by fNAV for the four ROIs corroborates this trend when considering the slope (m), intercept (b), and Pearson correlation coefficient (ρ) respectively along each spatial dimension (Figure 2C).

#### 3.1.2 ∣ Influence of respiratory motion on vessel area

**FIGURE 3:**
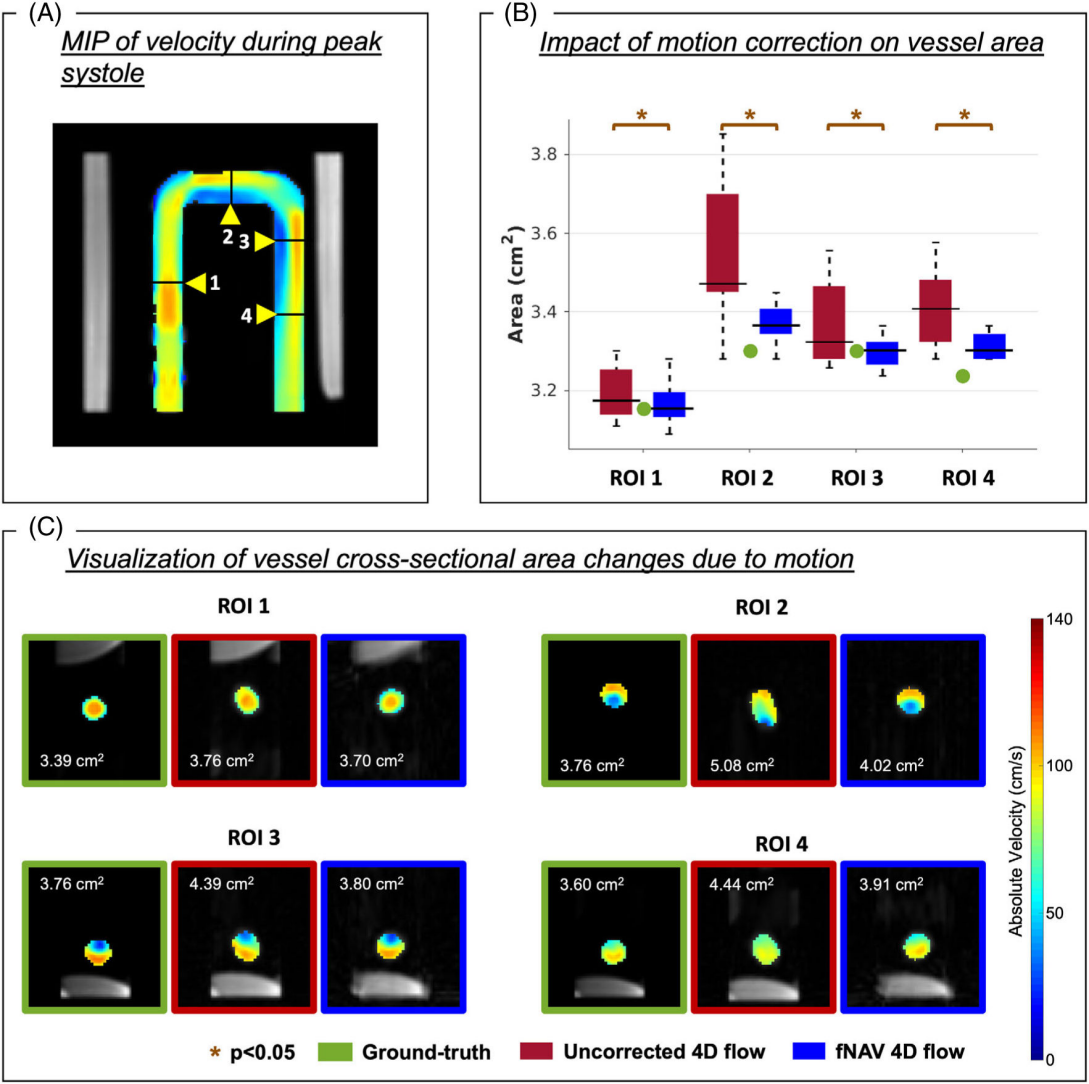
Influence of motion on the vessel area for four regions of interest (ROI). (A) Coronal view of the pulsatile flow phantom with the maximum intensity projection (MIP) of the velocity at peak systole shown and the four ROIs marked. (B) After focused navigation (fNAV) correction (blue), the area of each ROI became closer in agreement to the ground-truth ROI area (green), when compared to the uncorrected cases (red). (C) Cross-sectional visualization of the velocity in each ROI at peak systole for one example, showing ground-truth (green), uncorrected 4D flow (red) and fNAV 4D flow (blue) datasets. The area of each cross section is shown at the top left corner of each image. A visible vessel degradation is shown in uncorrected cases. In this example, the largest simulated motion displacement is shown for each ROI. *p*, *p*-value.

In general, the vessel area for each measured ROI in simulated radial 4D flow data is overestimated (Figure 3B) by uncorrected reconstructions because of motion blur, when compared to the ground-truth reference (Table 1). Conversely, fNAV 4D flow vessel area yielded a better agreement with the ground-truth reference area. This is further demonstrated by the differences in vessel area between uncorrected 4D flow and the ground-truth dataset, which were significantly higher than those measured between the fNAV 4D flow and the ground-truth datasets in all four ROIs (ROI 1: 0.11 ± 0.17 cm^2^ vs. 0.04 ± 0.14 cm^2^, ROI 2: 0.61 ± 0.43 cm^2^ vs. 0.18 ± 0.10 cm^2^, ROI 3: 0.15 ± 0.26 cm^2^ vs. 0.00 ± 0.09 cm^2^, ROI 4: 0.42 ± 0.22 cm^2^ vs. 0.18 ± 0.07 cm^2^, *p* < 0.05). The average vessel area difference between uncorrected and ground-truth 4D flow datasets (0.32 ± 0.11 cm^2^) was significantly different from the average vessel area difference between fNAV and ground-truth 4D flow datasets (0.10 ± 0.03 cm^2^, *p* < 0.05).

#### 3.1.3 ∣ Influence of respiratory motion on flow quantification

**FIGURE 4:**
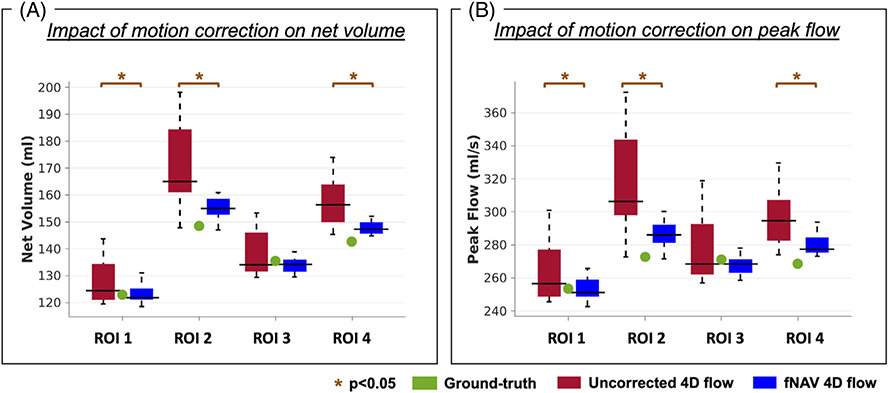
Influence of respiratory motion on flow quantification in a pulsatile flow phantom. Net volume (A) and peak flow rate (B) measurements calculated from focused navigation (fNAV) 4D flow and uncorrected 4D flow reconstructions of the simulated datasets (*n* = 20) across the same four ROIs shown in Figures 2 and 3. The ground-truth value for each ROI is marked in green and statistically significant differences between fNAV and uncorrected 4D flow measurements are denoted by orange stars.

Overall, simulated datasets with uncorrected respiratory motion resulted in flow measurements that were significantly different from the ground-truth values, whereas fNAV 4D flow datasets showed better agreement with the ground-truth. Still, the quantitative comparison of net volume and peak flow measurements showed different effects of motion across the 4 ROIs (Figure 4, Table 1).

Net volume average differences from the ground-truth were, for uncorrected and fNAV 4D flow datasets, respectively, 11.1 ± 3.5 mL vs. 2.6 ± 0.7 mL. Specifically, for ROI 2 and ROI 3, placed within the fNAV region, the net volume differences from the ground-truth were 22.4 ± 15.4 mL and 2.5 ± 8.3 mL for uncorrected 4D flow and 6.6 ± 3.7 mL and −1.6 ± 2.6 mL for fNAV 4D flow. ROI 1, located at the edge of the fNAV region, reported a net volume difference of 5.1 ± 8.4 mL for uncorrected 4D flow and 0.4 ± 3.8 mL for fNAV 4D flow. Net volume differences from ROI 4 were reduced from 14.3 ± 8.4 mL for uncorrected 4D flow to 5.0 ± 2.4 mL when correcting with fNAV. For peak flow, the same trend was reported (ROI 1: 11.0 ± 18.2 mL/s vs. −0.6 ± 6.9 mL/s; ROI 2: 43.9 ±29.5 mL/s vs. 13.4 ± 7.2 mL/s; ROI 3:6.6 ± 19.8 mL/s vs. −3.7 ± 5.1 mL/s; ROI 4: 27.6 ±15.7 mL/s vs. 11.2 ± 6.2 mL/s, uncorrected vs. fNAV 4D flow, respectively). The average difference in peak flow measurements was 22.3 ± 6.0 mL/s for uncorrected 4D flow and 5.1 ± 0.9 mL/s for fNAV 4D flow. Overall the average difference in net volume and peak flow measurements was significant between the two comparisons (*p* < 0.05). For individual ROIs, all flow measurements were significantly different between uncorrected and fNAV 4D flow datasets (*p* < 0.05), except in ROI 3 (*p* = 0.13 for net volume and *p* = 0.07 for peak flow).

### 3.2 ∣ Feasibility in a cohort of CHD patients

#### 3.2.1 ∣ 4D flow image reconstruction

Respiratory motion amplitudes estimated using fNAV were obtained for all patient datasets with a mean and SD of: A_*x*_ = 2.5 ± 2.0 mm, A_*y*_ = 2.5 ± 2.2 mm, A_*z*_ = 7.2 ± 3.6 mm, and ranging between A_*x*_ = 0.4–7.8 mm, A_*y*_ = 0–8.9 mm, A_*z*_ = 0.1–16.4 mm. These values are within the range of values tested in vitro.

**FIGURE 5:**
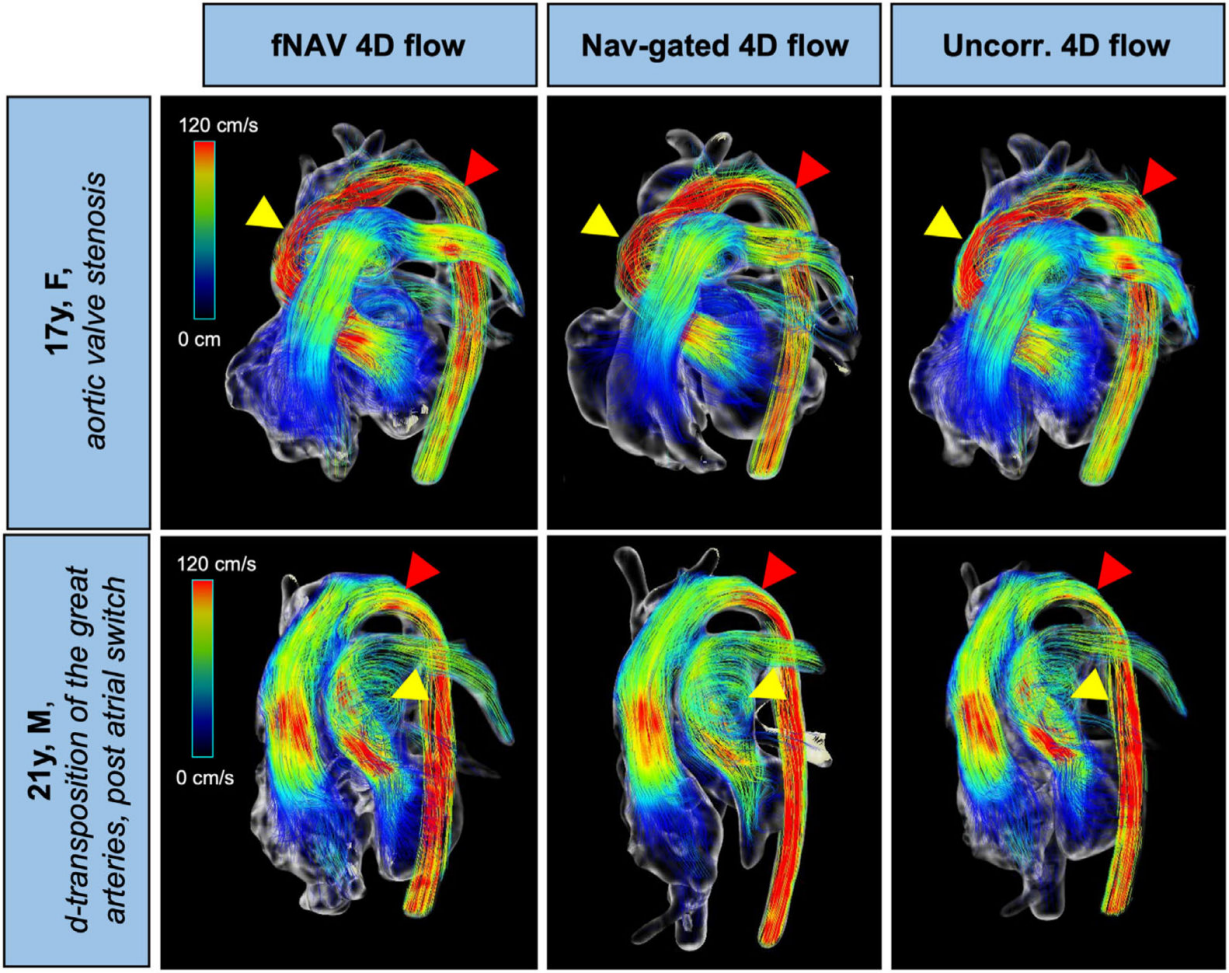
Visualization of flow streamlines in two patients with congenital heart disease using focused navigation (fNAV), nav-gated and uncorrected 4D flow datasets. Yellow arrows point to regions of large turbulence for each case, where image quality appears to be similar for both reconstructions. Red arrows point to regions with different flow streamlines, where the uncorrected 4D flow reconstructed dataset has larger differences to the nav-gated 4D flow dataset when compared to fNAV 4D flow. Uncorr. 4D flow, uncorrected 4D flow; F, female; M, male.

Streamline visualizations of all 4D flow datasets (fNAV, nav-gated, and uncorrected) are shown for two representative patients in Figure 5. Overall image quality is comparable between the three 4D flow datasets, including in regions of larger turbulence (yellow arrows). Red arrows highlight locations with small differences in the velocity streamlines between reconstructions, where the uncorrected 4D flow streamlines are visually the most different when compared to fNAV 4D flow and nav-gated 4D flow streamlines.

#### 3.2.2 ∣ Comparison of vessel area and flow measurements

Six MPA segmentations were excluded from the study, two because of poor visualization of the vessel from 2D flow datasets and four because of poor visualization overall, on all flow datasets. One nav-gated 4D flow dataset was corrupted and therefore, was also excluded from the analysis. From the remaining data, a total of 264 vessels segmented from all the 2D/4D flow datasets were included in the comparison.

**FIGURE 6:**
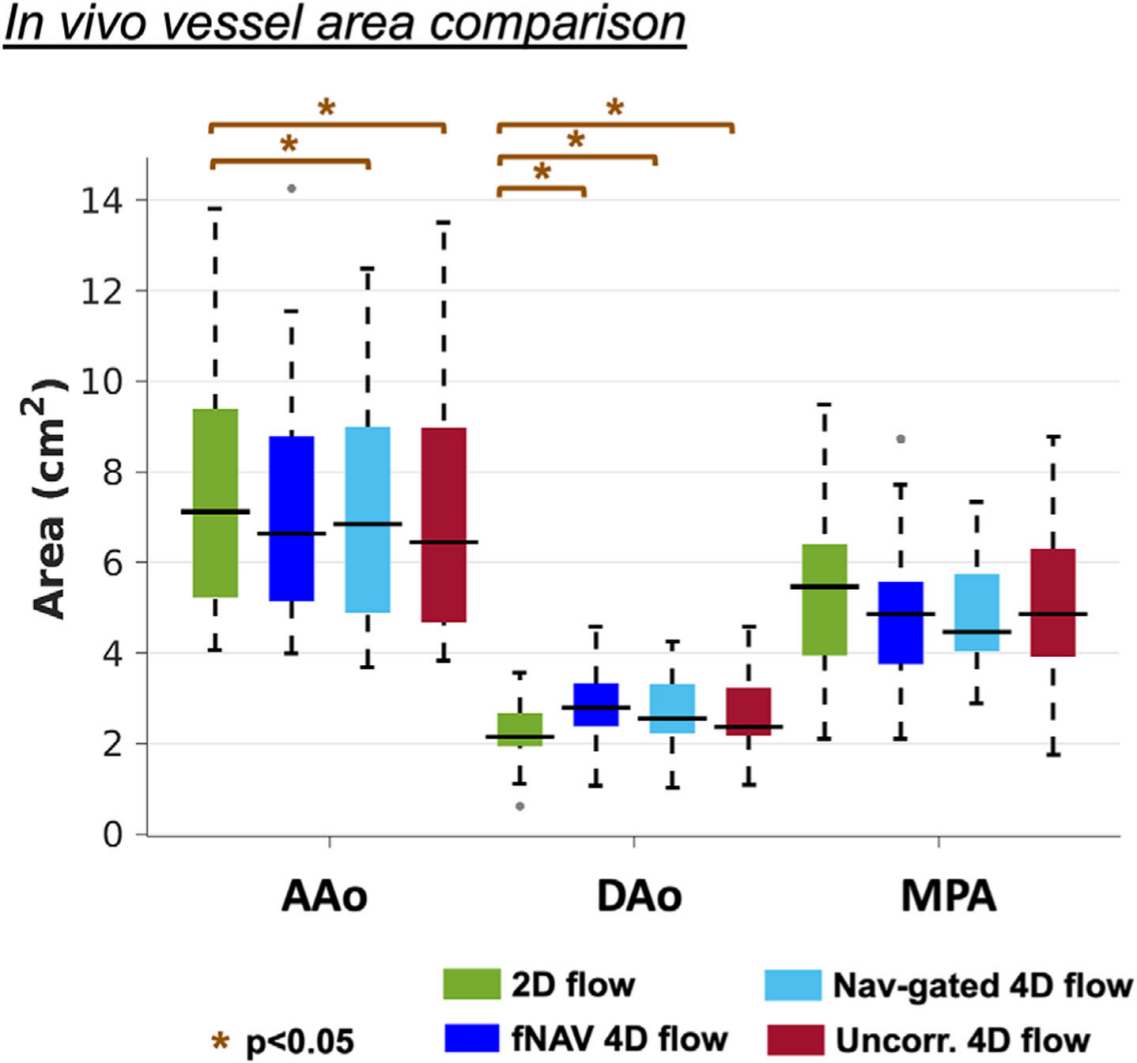
Comparison of vessel area from in vivo datasets. 2D flow (green), and focused navigation 4D flow (dark-blue), nav-gated 4D flow (light-blue) and uncorrected 4D flow (red) measurements are included for all patients. AAo, ascending aorta; DAo, descending aorta; MPA, main pulmonary artery; Uncorr. 4D flow, uncorrected 4D flow; *p*, *p*-value.

In the absence of a ground-truth measurement of vessel area, comparison between fNAV, nav-gated, and uncorrected 4D flow datasets to the reference 2D flow datasets (Figure 6) demonstrated variable agreement depending on the vessel of interest. The average vessel area measurements across all vessels were 4.92 ± 2.95 cm^2^ for 2D flow, 5.06 ± 2.64 cm^2^ for fNAV 4D flow, 4.87 ± 2.57 cm^2^ for nav-gated 4D flow, and 4.87 ± 2.69 cm^2^ for uncorrected 4D flow. No significant difference in AAo vessel area was found between fNAV 4D flow datasets (7.24 ± 2.63 cm^2^) and 2D flow datasets (7.39 ± 2.64 cm^2^, *p* = 0.38), but a significant underestimation as compared to 2D flow vessel area measurements was observed for nav-gated 4D flow (7.10 ± 2.50 cm^2^, *p* < 0.05) and uncorrected 4D flow (6.91 ± 2.64 cm^2^, *p* < 0.05). Conversely, DAo vessel area measurements from fNAV 4D flow (2.88 ± 0.85 cm^2^), nav-gated 4D flow (2.60 ± 0.77 cm^2^) and uncorrected 4D flow (2.57 ± 0.84 cm^2^) datasets were significantly (*p* < 0.05) overestimated relative to those from 2D flow datasets (2.22 ± 0.67 cm^2^). No significant differences were found in the MPA vessel area for fNAV (5.07 ± 1.72 cm^2^, *p* = 0.29), nav-gated (4.91 ± 1.31 cm^2^, *p* = 0.17) and uncorrected (5.24 ± 1.97 cm^2^, *p* = 0.74) 4D flow datasets when compared to 2D flow (5.25 ± 2.04 cm^2^).

**FIGURE 7:**
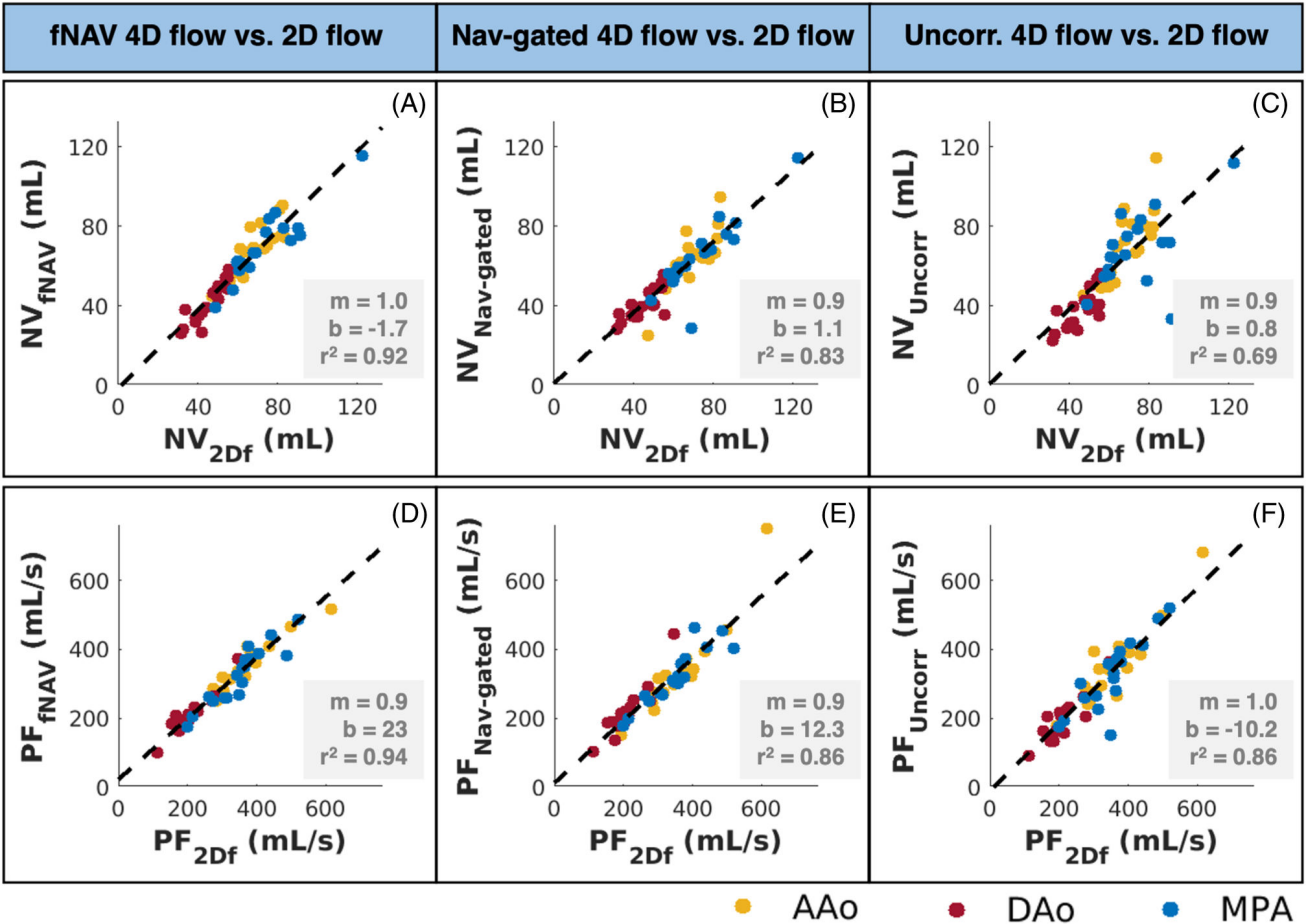
Linear regression of net volume (A–C) and peak flow (D–F) measurements between each 4D flow dataset and the reference standard 2D flow datasets. Overall, focused navigation (fNAV) 4D flow net volume and peak flow measurements (A and D) were the strongest correlated measures to 2D flow, when compared to nav-gated 4D flow (B and E) and uncorrected 4D flow (C and F) datasets. NV, net volume; PF, peak flow rate. *r*^2^, squared Pearson correlation coefficient. 2Df, 2D flow; fNAV, fNAV 4D flow; Nav-gated, nav-gated 4D flow; Uncorr, uncorrected 4D flow.

Linear regression results between 2D flow and fNAV 4D flow net volume measurements (*r*^2^ = 0.92) (Figure 7A) were overall the strongest correlation results when comparing 2D flow with other 4D flow datasets (*r*^2^ = 0.83 for comparison to nav-gated 4D flow) (Figure 7B) (*r*^2^ = 0.69 for comparison to uncorrected 4D flow) (Figure 7C). Similarly, 2D flow peak flow measurements had a larger correlation with fNAV 4D flow peak flow measurements (Figure 7D-F) (*r*^2^ = 0.94, *r*^2^ = 0.86, *r*^2^ = 0.86, respectively).

**FIGURE 8:**
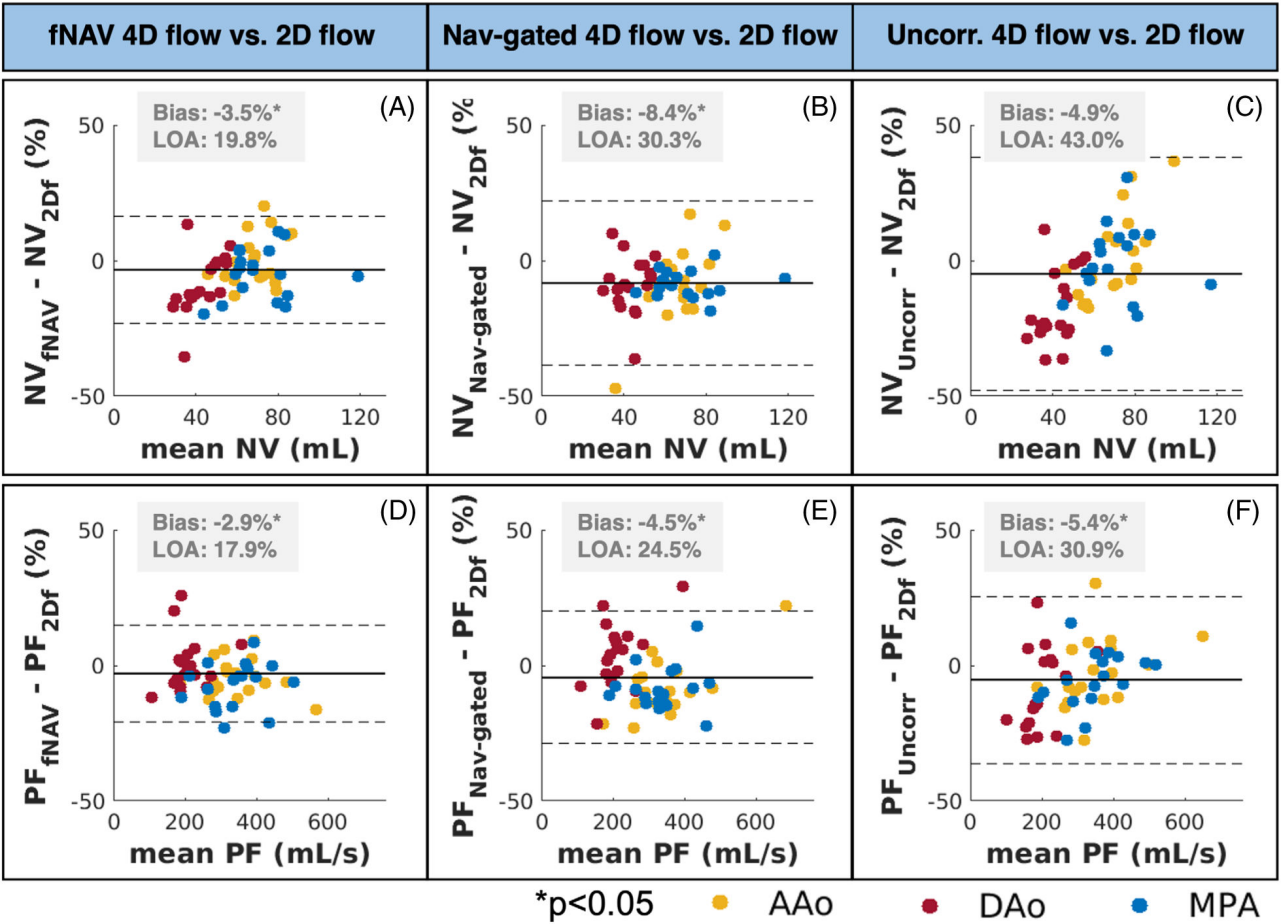
Bland–Altman results of net volume (A–C) and peak flow (D–F) measurements between each 4D flow dataset and the reference standard 2D flow datasets. Comparing to 2D flow datasets, focused navigation (fNAV) 4D flow net volume and peak flow measurements (A and D) had smaller biases compared to measures from nav-gated 4D flow (B and E) and uncorrected 4D flow (C and F) datasets. NV, net volume; PF, peak flow rate. LOA, limits of agreement. 2Df, 2D flow, fNAV, fNAV 4D flow; Nav-gated, nav-gated 4D flow; Uncorr, uncorrected 4D flow.

Compared to 2D flow, fNAV 4D flow datasets showed the lowest bias in net volume and peak flow across all 4D flow datasets (−3.5 ± 19.8% for net volume, −2.9 ± 17.2% for peak flow) (Figure 8), although all 4D flow net volume and peak flow measurements were significantly different from 2D flow measurements, with *p* < 0.05, with the exception of the net volume comparison between 2D flow and uncorrected 4D flow (−4.9 ± 43.0%, *p* = 0.06).

## 4 ∣ DISCUSSION

In this study, we extended the use of a previously described fNAV method for respiratory motion correction of free-running 3D radial PC-MRI acquisitions to obtain fNAV 4D flow datasets. The proposed fNAV 4D flow approach does not require image navigators or ECG-gating and can be acquired with simplified scan planning and a fixed scan time. Using fNAV, translational motion of the heart is estimated and used to correct all acquired readouts in radial 4D flow MRI. We validated the fNAV 4D flow framework in simulated data generated from non-rigid respiratory motion fields applied to a pulsatile flow phantom and demonstrated its feasibility in a cohort of patients with CHD. We successfully confirmed our hypotheses that: (1) fNAV can estimate respiratory motion from free-running 3D radial PC-MRI acquisitions, and that (2) the resulting fNAV 4D flow datasets produce comparable flow measurements to separately acquired reference measurements, and with a reduction in bias when compared to uncorrected 4D flow datasets. Although a regional variation in the accuracy of respiratory motion and resulting vessel area and flow measurements was observed because of the translational correction applied to a non-rigid underlying motion, overall fNAV 4D flow produced comparable results to the reference standard flow datasets and significantly improved uncorrected data.

### 4.1 ∣ Validation in a pulsatile flow phantom

In the first part of our study, we validated the use of fNAV for correction of respiratory motion in a numerical simulation based on data obtained from a pulsatile flow phantom. The data acquisition was performed without any physical displacement of the phantom during the scan. Instead, a retrospective approach was chosen to enable a well-controlled simulation of a large number of non-rigid displacements, which not only enabled the direct comparison of estimated fNAV coefficients with generated values, but it also enabled the direct comparison of all datasets induced with motion to the non-motion corrupted counterpart (ground-truth), removing any acquisition-related bias. This comparison demonstrated that respiratory motion impacts both the measured area of the vessel-like structure of the phantom and subsequent flow. These results corroborate previous studies, which also demonstrated a decrease in the accuracy of flow measurements and overall image quality when no respiratory compensation is considered.^21,22^

When using fNAV in vitro, the resulting vessel area and flow measurements demonstrated that fNAV can accurately measure and correct for rigid translational respiratory motion providing an overall improvement in accuracy relative to uncorrected data but with variation depending on the degree of underlying non-rigid motion. For instance, for ROI 2 and ROI 3, the estimated fNAV displacement measures matched with high correlation the generated displacements, enabling motion corrected 4D flow reconstructions that yielded area and flow measurements in strong agreement with the ground-truth, therefore, confirming our first hypothesis. Moreover, the estimated fNAV coefficients corrected the generated displacements simulated in ROI 1 and ROI 4 with lower accuracy, and, as a result, there were larger differences in the area of these ROIs when comparing fNAV 4D flow datasets to the ground-truth. These results are a consequence of using a rigid motion correction in a non-rigid structure. Nevertheless, the net volume and peak flow measures were still improved by applying motion correction, implying that motion correction, even if not perfect, is still beneficial when compared to the uncorrected counterpart. Nevertheless, at higher spatial resolution, these discrepancies may have greater impact on flow quantification.

The numerical simulation provided valuable insight on how the rigid fNAV correction of free-running 3D radial PC-MRI acquisitions would adapt to an *in vivo* setting, where motion is non-rigid. Still, increasing the complex-ity of the current phantom setup by including physical motion during the scan (for example with a moving cart) could help us further understand the limits of this technique for correcting respiratory motion in free-running 3D radial PC-MRI acquisitions. Additionally, a more rigorous validation in healthy volunteers that can be given instructions (i.e., periods of shallow or deep breathing) throughout the acquisition may be worth investigating in the future.

### 4.2 ∣ Feasibility in a cohort of CHD patients

To demonstrate the feasibility and evaluate the performance of the proposed fNAV 4D flow approach in vivo, we compared fNAV 4D flow datasets from a cohort of CHD patients to 2D flow datasets, nav-gated 4D flow datasets, and to uncorrected 4D flow reconstructions of the same data, in terms of vessel area and the resulting flow measurements.

The overall image quality obtained from fNAV 4D flow reconstructions in terms of the PC-MRA volumes and the velocity streamlines visualized at peak systole was comparable between the fNAV, nav-gated and uncorrected 4D flow datasets. Nevertheless, small differences in the velocity streamlines were observed (see Figure 5, red arrows). These discrepancies may have been caused by the anatomical segmentation, or because of SNR differences between acquisitions and reconstructions. However, given the lack of a ground-truth for comparison, understanding these subtle changes can be challenging.

In the absence of a ground-truth measurement, in vivo vessel areas from fNAV, nav-gated, and uncorrected 4D flow datasets were compared to 2D flow and yielded some discrepancies depending on the vessel of interest. These in vivo results are consistent with those from the numerical simulation where the agreement with respect to ground-truth varied according to the position of the ROI, suggesting that our in vivo results are also affected by the translational correction used in the presence of non-rigid motion. Nonetheless, the fNAV 4D flow vessel area showed better overall agreement with 2D flow than to nav-gated and uncorrected 4D flow datasets, again highlighting the fact that fNAV provides an accurate correction of rigid respiratory motion, but may be limited in areas more greatly impacted by the underlying non-rigid motion.

When comparing fNAV, nav-gated, and uncorrected 4D flow measurements of net volume and peak flow to the reference 2D flow, fNAV 4D flow had a lower bias and slightly improved limits of agreement. These results may suggest that the respiratory motion correction provided by fNAV is more representative of the breath-hold performed for the 2D flow acquisitions, when also compared to nav-gated 4D flow, as this technique usually includes a large acceptance window for respiratory motion.

In this proof-of-concept study, 3D translational respiratory-derived displacement of the heart was estimated with fNAV and used to correct the acquired k-space data. Although the corrected data yielded similar vessel segmentations and flow measurements to the reference 2D flow and nav-gated 4D flow acquisitions, the main limitation of the current approach is that it does not account for rotational motion or the more general non-rigid behavior of respiratory motion. In principle, additional fNAV coefficients could be included to account for rotational motion, and the same fNAV approach used in this work can be modified to perform non-rigid correction using the localized auto-focusing method as previously described.^23,25,39^ This approach is potentially prohibitive because of increases in computational time as separate reconstructions are required for different motion states, but this may be overcome by reconstructing the different motion states using parallel processing. Given the accurate performance of fNAV demonstrated in this work for specific regions, further investigation into the localized auto-focusing approach is warranted.

The current fNAV 4D flow approach could also be used to improve the quality of previously described respiratory motion-resolved 5D flow reconstructions^27^ by correcting translational intra-bin motion or could be combined with a more generalized strategy for inter-bin motion compensation using motion fields.^30,40-43^ Additionally, alternative strategies have been proposed to compensate rather than correct for respiratory motion in 4D flow MRI such as adaptive navigator gating^14,21,44,45^ and soft-gating,^18,39,46^ but may have limitations in the presence of significant respiratory motion or variability. As new advances in both acquisition and reconstruction methods enable higher spatial resolution, it is likely that a combination of these aforementioned methods including fNAV is needed to truly compensate for the complex non-rigid motion of the heart because of respiration and provide accurate estimations of blood flow. Regardless, of the potential improvements to and future applications of fNAV, validation of the method showing that it can accurately estimate respiratory motion and correct for it to produce high quality visualizations and quantifications of flow has been achieved in this work.

## 5 ∣ CONCLUSION

Respiratory motion correction for free-running 3D radial PC-MRI acquisitions has successfully been achieved using fNAV, both in a pulsatile flow phantom and in a cohort of patients with congenital heart disease. The resulting fNAV 4D flow datasets yielded accurate estimates of translational displacements of the heart because of respiration, resulting in similar vessel segmentations and flow measurements as those from both reference standard 2D flow and navigator-gated Cartesian 4D flow datasets. Using this approach, a quantitative evaluation of blood flow in the heart and its great vessels can be obtained in a fixed scan time, without the uncertainty in scan duration related to navigator efficiency, and without the need for respiratory navigators or ECG-gating.

## Supplementary Material

Table S1 **Table S1.** Patient cohort, age, gender, and diagnosis.

## Figures and Tables

**TABLE 1:** Mean and SD measurements for vessel area, net volume, and peak flow obtained for each of the four ROIs selected in the phantom.

Measurement	4D flow reconstruction	ROI 1	ROI 2	ROI 3	ROI 4
Vessel area (cm^2^)	Ground-truth	3.39	3.76	3.76	3.60
	Uncorrected	3.50 ± 0.17	4.37 ± 0.43	3.91 ± 0.26	4.02 ± 0.22
	fNAV	3.43 ± 0.14	3.93 ± 0.10	3.76 ± 0.09	3.77 ± 0.07
Net volume (mL)	Ground-truth	123.0	148.4	135.5	142.7
	Uncorrected	128.1 ± 8.4	170.9 ± 15.4	137.9 ± 8.3	157.1 ± 8.4
	fNAV	123.4 ± 3.8	155.1 ± 3.7	133.8 ± 2.6	147.7 ± 2.4
Peak flow (mL/s)	Ground-truth	253.7	273.1	271.3	268.6
	Uncorrected	264.7 ± 18.2	317.0 ± 29.5	277.9 ± 19.8	296.3 ± 15.7
	fNAV	253.1 ± 6.9	286.4 ± 7.2	267.6 ± 5.1	279.9 ± 6.2

*Note*: The reference 4D flow reconstruction (without motion) is used as ground-truth for comparison with 4D flow reconstructions of different respiratory motion displacements without (uncorrected 4D flow) and with (fNAV 4D flow) motion correction.

Abbreviations: ROI, region of interest; fNAV, focused navigation.
