# Supplementary material for: Focused navigation for respiratory–motion-corrected free-running radial 4D flow MRI
Source: Magn Reson Med. Author manuscript; Available in PMC 2024 Jan 1. (PMC10149606; doi:10.1002/mrm.29634)
Supplement: Table S1 — Patient cohort, age, gender, and diagnosis. [file NIHMS1890196-supplement-Table_S1.docx]

# Supplementary Material

| Age | Gender | Diagnosis |  |
| --- | --- | --- | --- |
| 55 | F | Turner syndrome with dilatation of the ascending aorta | |
| 29 | F | Coarctation of the aorta, status-post resection and end-to-end anastomosis | |
| 21 | M | d-transposition of the great arteries, status-post atrial switch (Senning) | |
| 21 | M | Pulmonary atresia with ventricular septal defect, status-post correction with a pulmonary valved conduit and a Melody valve | |
| 27 | F | Status post pulmonary valvuloplasty with pulmonary regurgitation | |
| 21 | M | Corrected total anomalous pulmonary venous return | |
| 35 | F | Turner, partial anomalous pulmonary venous return | |
| 33 | F | Tetralogy of Fallot, status-post repair with transannular patch | |
| 18 | M | Bicuspid aortic valve, aortic insufficiency | |
| 19 | M | Tetralogy of Fallot, status-post repair with transannular patch | |
| 39 | M | d-transposition of great arteries, status-post atrial switch | |
| 21 | F | Ross Operation for aortic stenosis | |
| 25 | M | Ross Operation for aortic stenosis | |
| 26 | M | pulmonary atresia, stenosis | |
| 17 | F | Patent ductus arteriosus | |
| 14 | M | Ebstein's anomaly, status-post cone reconstruction | |
| 17 | F | Aortic valve stenosis (bicuspid), status-post valvuloplasty | |
| 7 | M | Hypoplastic left heart syndrome, status post Fontan | |
| 19 | M | Ross Operation for aortic stenosis | |
| 52 | M | Ross Operation for aortic stenosis | |
| 22 | F | Ebstein’s anomaly, staus-post De Vega annuloplasty and Glenn anastomosis | |
| 60 | F | Tetralogy of Fallot, Status-post correction surgery | |
| 22 | M | Ross Operation for aortic stenosis | |
| 10 | M | d-Transposition of the great arteries, status-post atrial switch | |
| 54 | M | d-Transposition of the great arteries, status-post atrial switch | |

**Table S1. Patient cohort, age, gender and diagnosis.** The cohort for this study was largely heterogeneous, to account for different anatomical structures when validating 4D flow fNAV.
